# Agreement between objective and subjective definitions of inactive disease, remission and minimal disease activity in juvenile idiopathic arthritis

**DOI:** 10.1186/1546-0096-9-S1-P197

**Published:** 2011-09-14

**Authors:** B Schiappapietra, A Consolaro, S Magni-Manzoni, N Solari, S Lanni, S Davì, S Pederzoli, G Bracciolini, A Martini, A Ravelli

**Affiliations:** 1 Pediatria II, IRCCS G.Gaslini, Genova, Italy; 2 Dipartimento di Pediatria, Università degli Studi di Genova, Italy; 3 Clinica Pediatrica, Fondazione IRCCS Policlinico S. Matteo, Pavia, Italy

## Background

In juvenile idiopathic arthritis (JIA), substantial disagreement between physicians, parents and children over disease remission can lead to difficulty in assessing the efficacy of treatments. It is, therefore, important to ascertain whether clinicians’, parents’ and children’s opinions converge and diverge and whether the recently developed criteria for inactive disease (ID) or minimal disease activity (MDA) may help enhance concordance.

## Objective

To evaluate the concordance between established (“objective”) criteria for ID (1) and MDA (2) and physician’s, parent’s and child’s “subjective” assessment of remission in children with JIA.

## Methods

A total of 618 consecutive children underwent the assessment of the traditional physician-reported, parent-reported, and child-reported outcome measures in a total of 1779 visits. Furthermore, at each visit the physician, a parent and the child were asked to judge independently and subjectively whether the disease was in remission or not. The first visit in which the criteria for ID and MD were met or that was judged as corresponding to a state of disease remission by the physician, the parent or the child was identified. Concordance between objective definitions of ID and MDA and subjective definitions of remission was examined by means of kappa statistics (<0.40=poor agreement; 0.41-0.60=moderate agreement; 0.61-0.80=substantial agreement; >0.80 excellent agreement).

## Results

The level of agreement (kappa values) between definitions is reported in table 1.

**Table 1:** 

	Inactive disease	Child remission	Parent remission	Physician remission
Minimal disease activity	0.66	0.54	0.58	0.72
Physician remission	0.64	0.53	0.62	-
Parent remission	0.48	0.87	-	-
Child remssion	0.45	-	-	-

## Conclusion

There was substantial agreement between physician’s subjective rating of remission and the presence of MDA and ID, and between physician’s and parent’s subjective rating of remission. Agreement between parents and children was excellent. Agreement was poorest between parent’s and child’s subjective ratings of remission and the presence of ID.

